# Thick Film Ni_0.5_Mn_0.5−x_Sn_x_ Heusler Alloys by Multi-layer Electrochemical Deposition

**DOI:** 10.1038/s41598-018-29628-8

**Published:** 2018-08-09

**Authors:** Yijia Zhang, Patrick J. Shamberger

**Affiliations:** Department of Materials Science and Engineering, Texas A&M University, College Station, TX 77843 USA

## Abstract

The design of multifunctional alloys with multiple chemical components requires controllable synthesis approaches. Physical vapor deposition techniques, which result in thin films (<1 μm), have previously been demonstrated for micromechanical devices and metallic combinatorial libraries. However, this approach deviates from bulk-like properties due to the residual stress derived in thin films and is limited by total film thickness. Here, we report a route to obtain ternary Ni-Mn-Sn alloy thick films with controllable compositions and thicknesses by annealing electrochemically deposited multi-layer monatomic (Ni, Mn, Sn) films, deposited sequentially from separate aqueous deposition baths. We demonstrate (1) controllable compositions, with high degree of uniformity, (2) smooth films, and (3) high reproducibility between film transformation behavior. Our results demonstrate a positive correlation between alloy film thicknesses and grain sizes, as well as consistent bulk-like transformation behavior.

## Introduction

Multifunctional alloys, are of interest due to the intrinsic ability of these materials to perform work, including mechanical actuation (e.g., one-way and two-way shape memory effects, magnetic shape memory effects) and heat pumping or refrigeration cycles (e.g. magnetocaloric effect, elastocaloric effect)^1,2^. These alloys show diverse magnetic and electric phenomena based on martensitic phase transformations between the high-temperature, high symmetry (cubic) phase and low-temperature, low symmetry phase, which can be induced by strain^3^, pressure^4^, magnetic field^5,6^, or other external thermodynamic driving forces.

Decreasing the length-scale of multifunctional alloys enables new functionality necessary for certain applications. (1) Integration with microelectronic and micromechanical systems. For example, Juan *et al*. developed nanoscale Cu-Al-Ni shape-memory alloys with ultrahigh damping performance that could help them to integrate into small/efficient micro electro-mechanical systems (MEMS)^7^. (2) Rapid heat transfer through caloric alloy films to some heat transfer fluid. For example, binary Ti-Ni and quaternary Ti-Ni-Cu-Co elastocaloric films in the micrometer thickness range result in rapid heat transfer^8^. (3) Development of strain-coupled magnetoelectric composites. For example, microscale Tb–Dy–Fe alloy particles were filled in PZT/polymer mixture to produce three-phase multiferroic composites, which resulted in a strong magnetoelectric response at low frequencies^9^. (4) Design of microstructured multifunctional foams and composites. For example, Queheillalt *et al*. reported the synthesis of stochastic Ni-based foams on carbon foam templates^10^. To use multifunctional alloys in these applications, we need a method to controllably deposit ternary/quaternary alloy films across a relevant range of length-scales (<1 µm to 100 µm).

Thin films of multifunctional alloys from microscale to nanoscale have been successfully produced by physical vapor deposition (PVD), techniques including evaporation and sputtering, in which differs by the average kinetic energy of atomic species being deposited. These PVD techniques have been applied to make epitaxial films^11^, polycrystalline films^12^, and nanoparticles of multifunctional alloys^13^. Also, high-throughput thin film composition spread technique using magnetron co-sputtering has been explored for combinatorial materials processing and development of material libraries^14–16^. However, one key limitation of PVD is the tendency for intrinsic properties of thin films (e.g. transformation temperature and hysteresis) to deviate significantly from bulk properties^17^. This phenomenon is caused by two main factors, surface energy effects and residual film stress^18^. Another important disadvantage is the limitation in film deposition rate, which practically limits overall film thickness over a certain critical value (generally <1 µm). Thus, it is difficult to attain desired bulk-like properties in a specific thin film, or to extrapolate observed thin film properties to a particular bulk alloy composition. Finally, deposition of films in complex geometries (i.e., not flat films) from most PVD approaches is challenging.

Electrochemical deposition (ED), as a controllable film deposition technique, has the ability to deposit either thin or thick films with high purity, low surface roughness, and controllable compositions and microstructures. Due to these advantages, electrochemical deposition has been adopted in the semiconductor industry for the deposition of chalcogenide and oxides^19,20^, the hard-disk drive industry for the deposition of magnetic recording heads^21^, and the corrosion and biomedical sectors for the deposition of biodegradable implant materials^22,23^. Compared with PVD technique, ED technique has three principle advantages. (1) ED is a relatively cost-effective deposition processes, requiring simple equipment, as compared with high vacuum chambers required for PVD processes. (2) Electrochemical deposition rates are in the range of (0.001 to 1) µm/s, up to three orders of magnitude faster than physical vapor deposition rates^24^. (3) ED shows greater control of residual stresses in the deposited film by near-room temperature deposition and growing thicker (>10 µm) films to minimize intrinsic internal film stresses^18,25^. On the contrary, the principle disadvantages of ED technique are the necessity of an electrically conductive substrate, and the limited compositional range of films. Highly reactive or refractory metals, such as Ti, Hf, and Zr, are difficult to deposit using aqueous ED technique due to their larger negative redox potential than hydrogen^26^. Significant effort is focused on developing ionic liquids to allow for the direct electrochemical deposition of reactive metals^27^.

Electrochemical deposition of multi-component alloys through sequential deposition of monatomic films results in greater compositional control than single-pot co-deposition approaches. Co-deposition of multi-component alloys from a single deposition bath has previously been demonstrated, including Ni-Mn, Ni-P, Zn-Ni-Co, Cu-In-Ga-Se, etc., but results in only limited control of alloy compositions^28–35^. Although efforts to exert greater compositional control have been put forward, including the adjustment of electrolyte concentration, current density, electrode rotation rate, etc., it remains challenging to achieve alloys with arbitrary composition by the single-layer co-deposition approach^36–38^. In contrast, Gaitzsch *et al*. produced Ni-Mn-Ga alloys by annealing individually deposited Ga, Mn, and Ni layers^39^. This triple-layer film deposition method, followed by thermal annealing, decouples the deposition of individual components, allows for simultaneous control over composition and film thickness.

In this study, we report a route to obtain thick-film Ni-Mn-Sn alloys with bulk-like transformation behavior by annealing multi-layer films following an experimentally determined sequence. The total number of layers is adjusted from three-layer (W//Mn/Sn/Ni) to thirty-layer (W//[Mn/Sn/Ni]/[Mn/Sn/Ni]/[Mn/Sn/Ni]…) by repeated stacking of 3n individual monatomic films. The overall film compositions and thicknesses are controlled by adjusting each layer thickness based on deposition time. Following ED, we thermally anneal films under a reducing gas to chemically homogenize films, resulting in recrystallization of the ternary Heusler alloy phase. We directly address the challenge of annealing films to promote compositional homogeneity, while minimizing film oxidation and mass loss. Decreasing grain sizes of a series of films with decreasing thicknesses are presented. This deposition approach allows for a platform for detailed investigation of the role of dimensionality in reversible martensitic phase transformation in multifunctional alloys, as well as a potentially scalable approach to grow thick-film Heusler alloys.

## Results

### Deposition of Monatomic Films

#### Manganese Deposition

Because of the low potential of Mn in aqueous solutions (E_0_[Mn^2+^/Mn] = −1.18 V_SHE_), Mn is generally regarded as the most electronegative metal which can be electrochemically deposited from aqueous solution^40^. Cyclic voltammetry of manganese deposition was analyzed to investigate Mn oxidation and reduction processes in the aqueous solution (Fig. S1a). Upon sweeping the potential from (−1.9 to −2.2) V, the magnitude of the current density increased precipitously from 4 mA/cm^2^ to 25 mA/cm^2^. Both the hydrogen evolution reaction (HER) and the manganese electrodeposition reaction (MEDR) were observed by the generation of bubbles and formation of a silvery metallic film on the cathode surface. Upon positively scanning from (−2.2 to 2) V, the Mn oxidation peak occurs beginning at −0.5 V, which was interpreted as the oxidation of the freshly deposited Mn at the cathode^41^. During electrochemical deposition, current efficiencies have been previously reported to increase to above 70% at 65 mA/cm^2^, then decreased gradually as current densities increased to 100 mA/cm^2^, accompanied by an increase in surface roughness^40^. Thus, a current density of 80 mA/cm^2^ was selected to obtain smaller grain sizes, while not decreasing current efficiency beyond acceptable limits. The deposition rate increased to 1.03 mg/min (1.46 μm/min) at the second minute and behaved linearly until ten minutes. The current efficiency was 79%, which was calculated by Equation (1), where *m* (g) was the deposited weight at a current *I* (A), *t* (s) was the deposition time, *M* was the atomic mass, *n* was the electron number, *F* was the Faraday constant^42^. The low current efficiency was because of the hydrogen evolution reaction on the cathode surface. The abrupt decrease of deposition rate after ten minutes was attributed to the reduced counter electrode surface area, which was coated by black manganese oxide during the electrochemical deposition process in the one compartment electrochemical cell. Double compartment electrochemical cells were designed for solving this problem^41^. We chose to use the simple one compartment electrochemical cell because we can obtain expected thickness before the counter electrode surface area was reduced.

1 $$\eta =\frac{mnF}{ItM}$$

**Figure 1:**
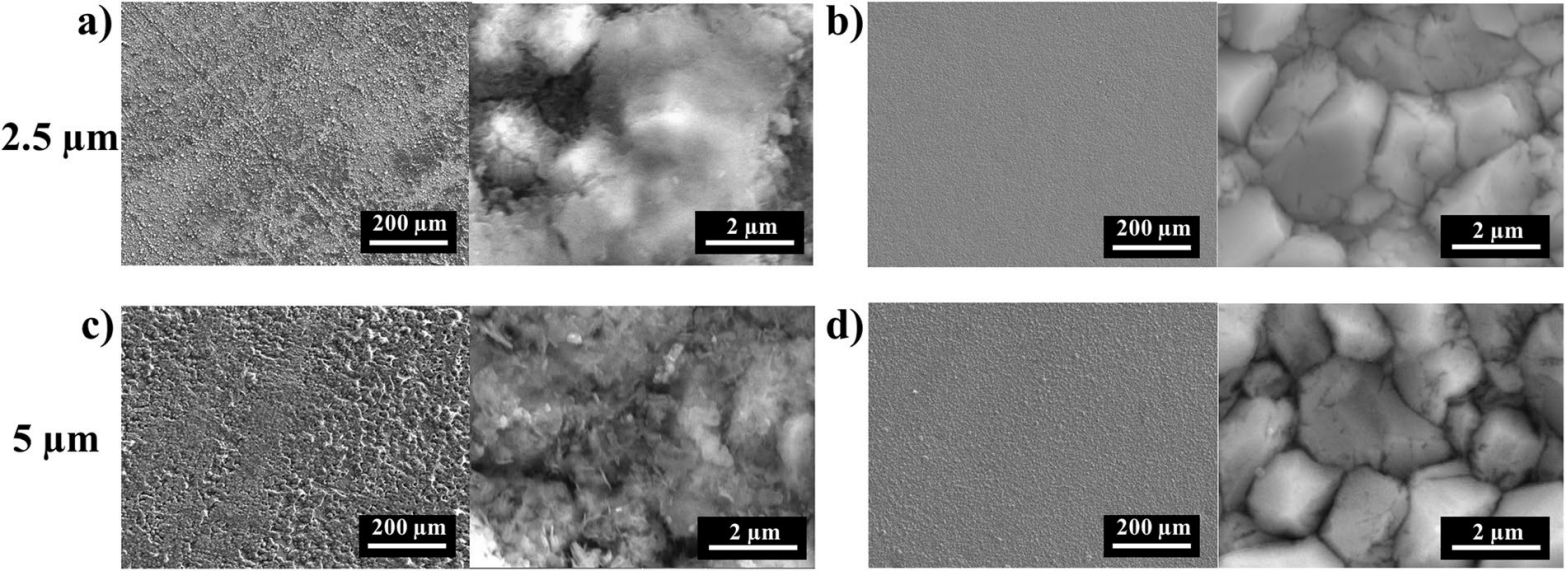
Electrochemical deposition of 2.5 μm thick Mn film on (**a**) as-received, and (**b**) polished W and 5 μm thick Mn film on (**c**) as-received, and (**d**) polished W substrates.

Mn films of two thicknesses (2.5 and 5 μm) were deposited on as-received and mechanically polished W substrates. The polished W substrate (areal root mean squared roughness, S_q_ = 5.3 ± 0.9 nm) was significantly smoother than the as-received W (S_q_ = 15.9 ± 2.0 nm); Fig. S2. Both 2.5 and 5 μm thick Mn films deposited on as-received W substrates were noticeably rougher than those deposited on polished substrates (Fig. 1). Mn films deposited on the polished W substrates were formed by the close growth of polygonal grains with average grain size near 2.3 μm (Fig. 1b,d). Furthermore, as-received substrates increased the contact area with air which resulted in significant manganese oxidation (25.3 wt % O in Mn film). Mn films deposited on polished W substrates contained ≤1.5 wt% O, as measured by EDS. In order to avoid the influence from HER, we tested the effect of adding ammonium hydroxide to adjust the solution to pH 6. The formation of ammonium sulfate worked as a buffer, preventing precipitation of manganese hydroxides to some degrees^43,44^. However, the resulting Mn films had little difference with the films which were deposited in the solution without ammonium hydroxide (Fig. S3) and thus, ammonium hydroxide was not used in Mn deposition bath solutions for the remainder of the study.

#### Nickel Deposition

**Figure 2:**
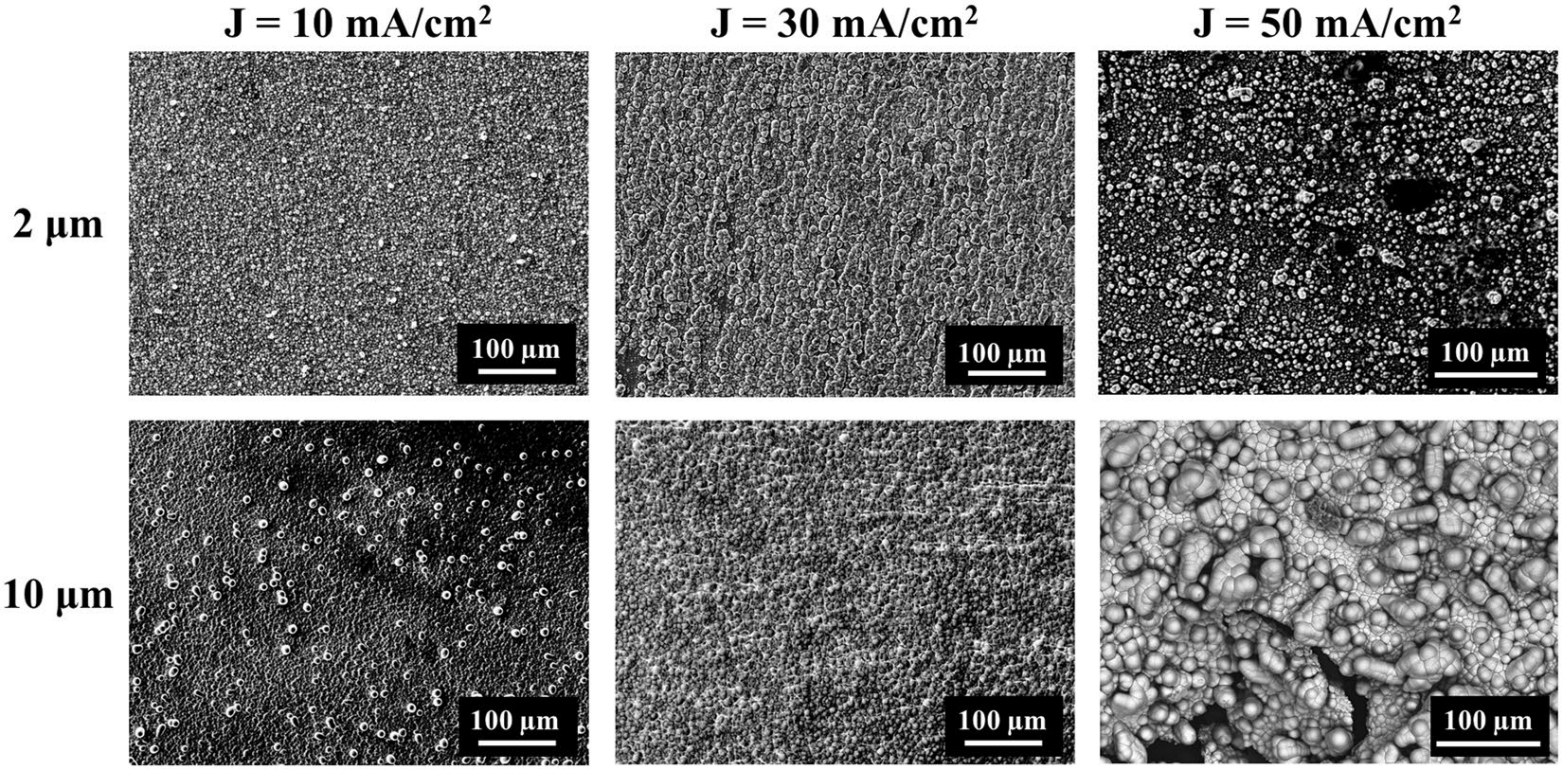
Electrochemical deposition of (2 and 10) μm thick Ni films at current densities of 10 mA/cm^2^, 30 mA/cm^2^, and 50 mA/cm^2^.

Nickel film reduction and oxidation processes were explored by cyclic voltammetry (Fig. S1b). Ni^2+^ was reduced at negative potential from (−0.7 to −1.1) V, leading to deposition of metallic films, and Ni films were oxidized at positive potential from (−0.4 to −0.1) V^45^. Morphologies of Ni films deposited at current densities of 10, 30, and 50 mA/cm^2^ were compared for (2 and 10) μm thick films (Fig. 2). Ni film deposited at a current density of 50 mA/cm^2^ had a rough surface morphology which was consisted by heterogeneous globular grains with grain size in the range of near (2 to 18) μm. By decreasing the current density to 30 mA/cm^2^, a smooth nickel film with average grain size of 4.6 μm was deposited at a rate of 0.49 mg/min (0.58 μm/min) with ≤1.2 wt% O. The current efficiency was 94%. Further decreasing current density to 10 mA/cm^2^ caused isolated Ni particles to grow on the surface in thicker films, and increased the overall film roughness.

#### Tin Deposition

**Figure 3:**
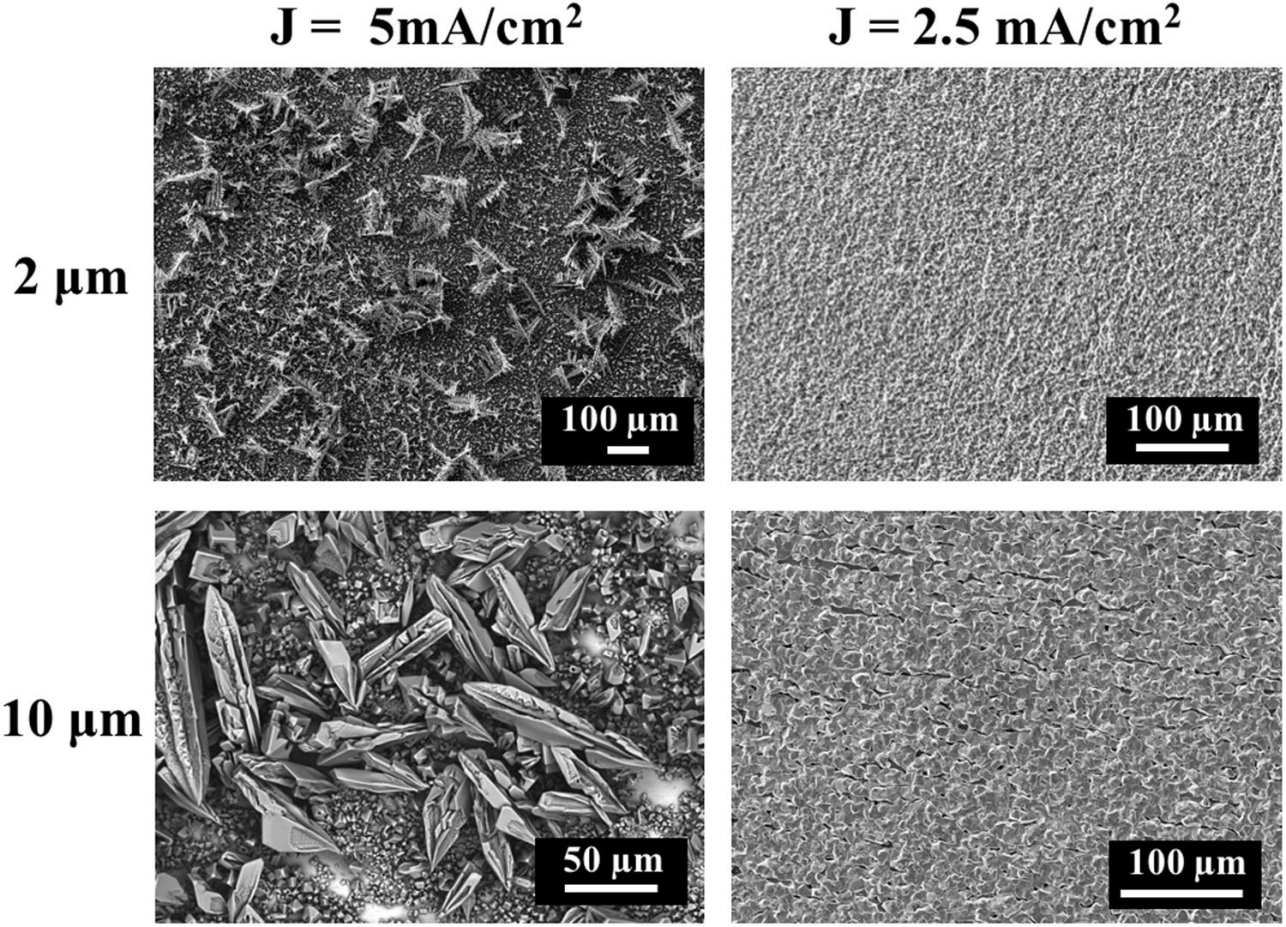
Electrochemical deposition of (2 and 10) μm thick Sn films at current densities of (2.5 and 5) mA/cm^2^.

Tin electrochemical deposition voltages and current densities were explored by cyclic voltammetry (Fig. S1c). Current densities of (5 and 2.5) mA/cm^2^ were used to plate (2 and 10) μm thick Sn films. Sn films deposited at a current density of 5 mA/cm^2^ adopted a dendritic structure larger than 30 μm, resulting in very high surface roughness, whereas decreasing current density to 2.5 mA/cm^2^ resulted in a plating rate of 0.08 mg/min (0.12 μm/min) and promoted a smoother tin film deposition (Fig. 3). The current efficiency was 91%. Comparing (2 and 10) μm thick films (2.5 mA/cm^2^), thickness did not have a noticeable influence on film roughness.

### Multi-layer Films

Thick alloy films with the desired composition (Ni_0.5_Mn_0.5−x_Sn_x_) were obtained by depositing multiple elemental layers, followed by an annealing step to chemically homogenize the film. The sequence of film deposition was determined by considering film adhesion and oxidation. For the bottom layer in contact with the substrate, the low adhesion of Ni film on the W substrate (W//Ni) led to delamination of the Ni film from the W substrate during deposition. The low melting temperature of Sn film (505 K) led to similar delamination of Sn film from a W substrate (W//Sn) during the annealing step. Thus, Mn was generally selected as the first electrochemically deposited layer on the W substrate (W//Mn). Also of consideration, Mn film was very susceptible to oxidation, and thus was preferably not exposed as the uppermost layer. The deposition sequence W//Mn/Sn/Ni and repeat stacking of this sequence (W//[Mn/Sn/Ni]/[Mn/Sn/Ni]/[Mn/Sn/Ni]…) led to the most repeatable film deposition. Gaitzsch *et al*. used a similar deposition sequence (W//Mn/Ga/Ni) because of these reasons^39^.

Following the previous portion of the study, current densities of 80 and 30 mA/cm^2^ were used for plating Mn and Ni films. For the Sn layer, dendritic structures occurred at low deposition currents when depositing on Mn layer (W//Mn/Sn). Thus, the current density for plating Sn films was decreased to 1 mA/cm^2^ and the concentration of the Sn deposition solution was decreased to 0.03 M SnSO_4_ and 0.07 M C_6_H_5_Na_3_O_7_ during the multiple elemental deposition. Triplet layers W//[Mn/Sn/Ni]_n_, where *n* is the total number of triplets, were deposited on polished W substrates. Throughout this study, overall thicknesses of multi-layer films were held constant at 28.9 µm, unless otherwise stated.

**Figure 4:**
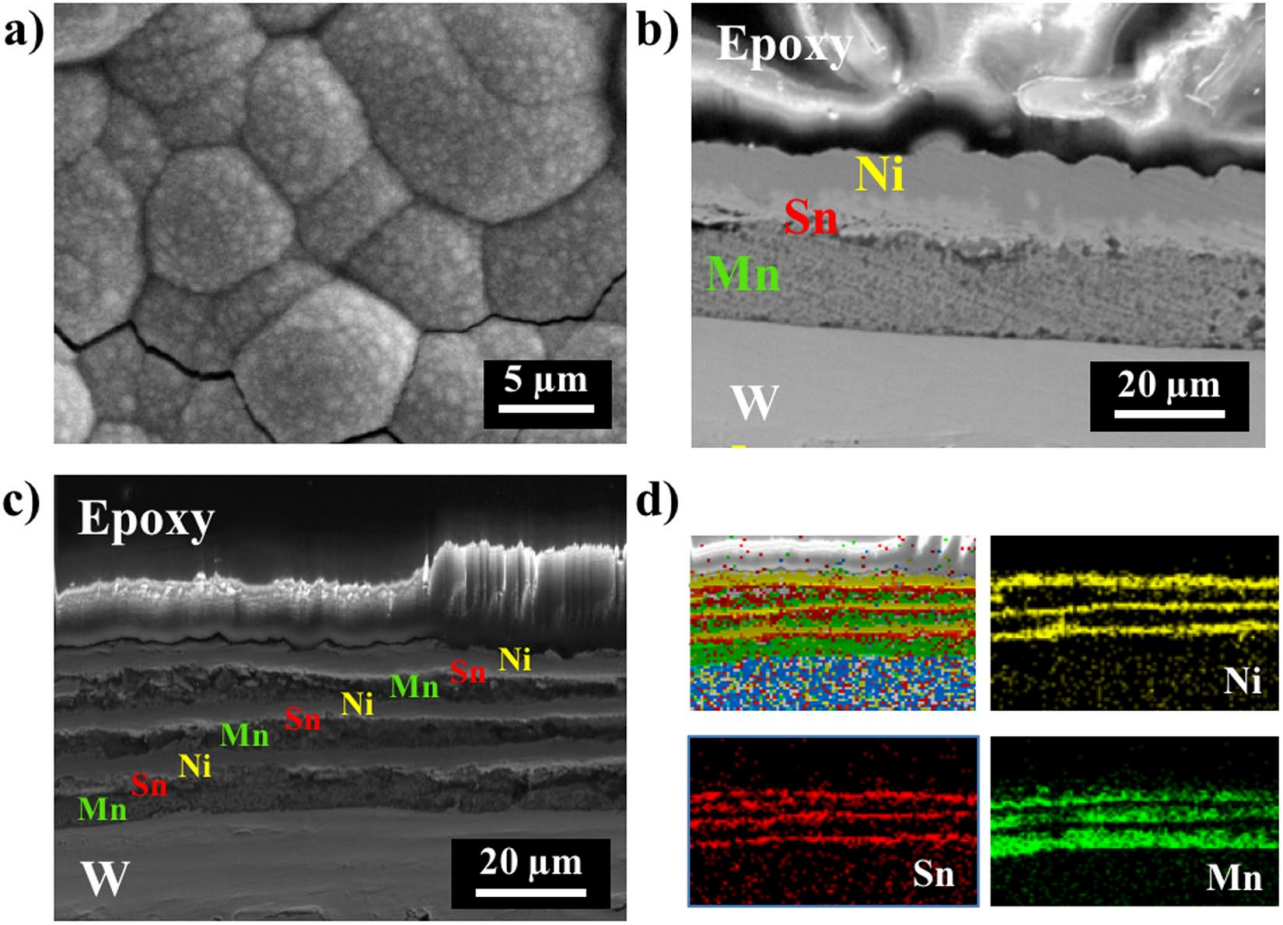
(**a**,**b**) Single triplet (*n* = 1) and (**c**,**d**) three triplet (*n* = 3) of Ni_0.5_Mn_0.369_Sn_0.131_ film before annealing. (**a**) Topography of film surface. (**b**,**c**) SEM image of film cross-sections, and (**d**) Ni (yellow), Sn (red), and Mn (green) compositional analysis by EDS.

The surface micrograph and cross-section images of triple-layer and nine-layer films under SEM showed the smooth film surface and uniform film thickness that laid the foundation for the following homogeneous alloys (Fig. 4a–c). EDS identified Mn, Sn, and Ni elements, and illustrated successful deposition of repeated stacking of the 3n-layer films (Fig. 4d). The desired composition Ni_0.50_Mn_0.37_Sn_0.13_ (x = 0.13) was verified by EDS (Fig. S4).

### Annealed Ternary Alloy Films

#### Annealing Conditions

**Figure 5:**
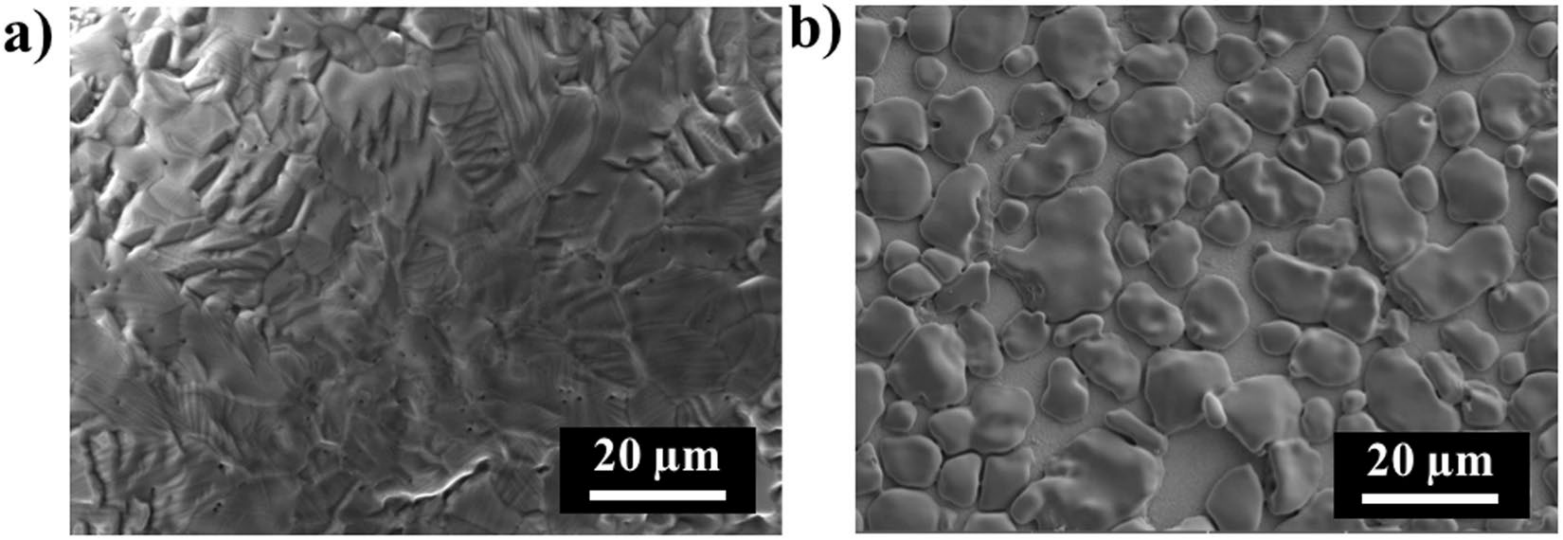
Surface micrographs of the triple-layer films after annealing at (**a**) 1073 K, 6 hours illustrating surface textures representative of incomplete homogenization and (**b**) 1273 K, 6 hours illustrating surface textures of significant mass loss.

**Figure 6:**
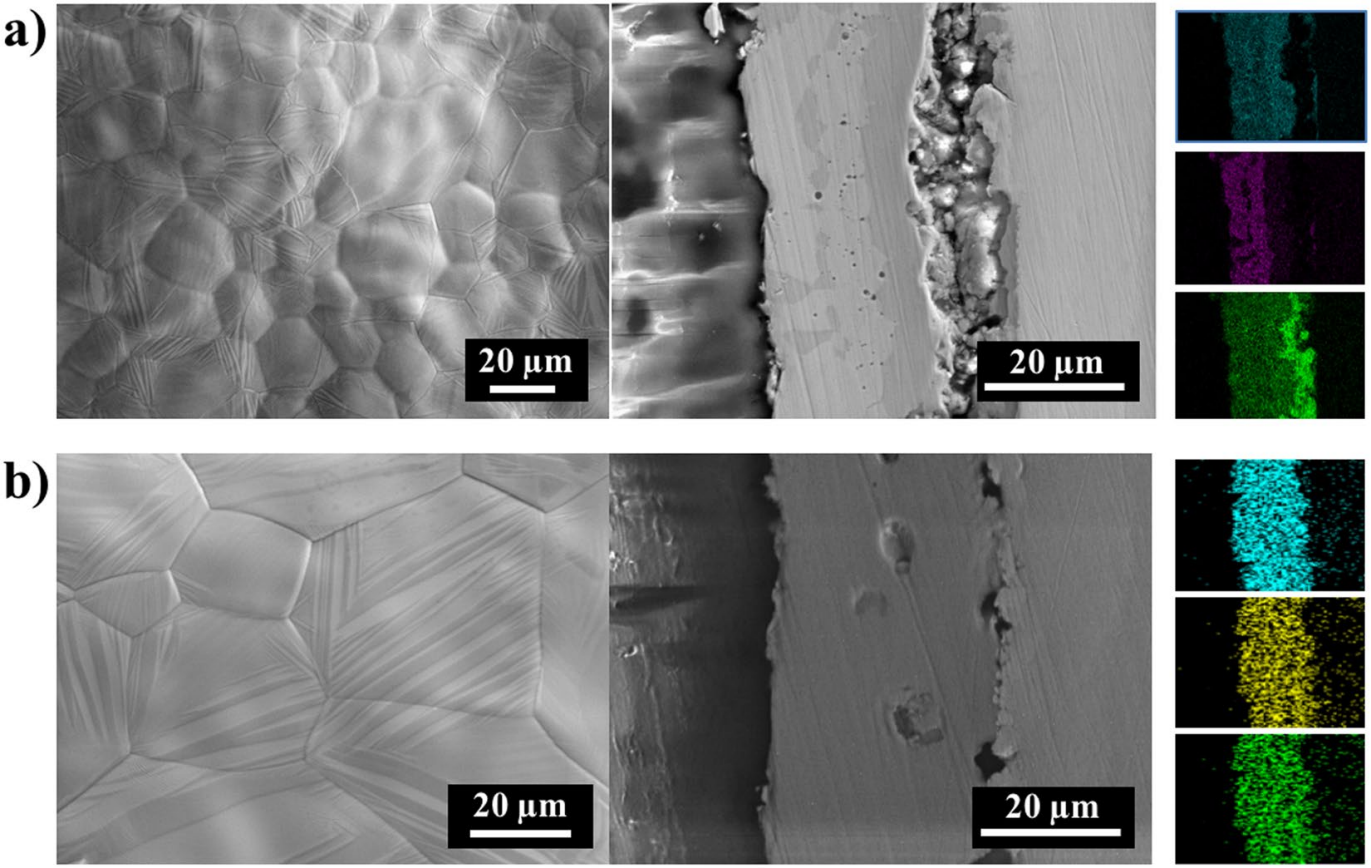
SEM images of surface and images and EDS mapping of cross-sections of (**a**) the (*n* = 1) film and (**b**) (*n* = 3) film after annealing 1273 K, 1.5 hours.

**Figure 7:**
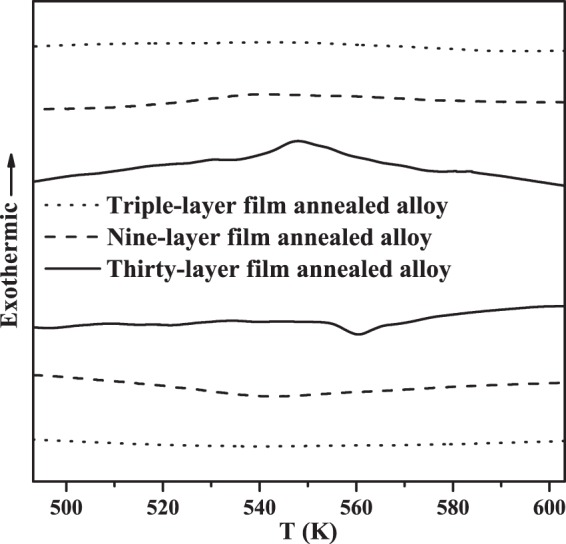
The DSC measurements of triple-layer (*n* = 1, 1.0 μm), nine-layer (*n* = 3, 8.7 μm), and thirty-layer (*n* = 10, 28.9 μm) film annealed alloys (Ni_0.50_Mn_0.419_Sn_0.081_).

Electrochemically deposited layered films were annealed under reducing conditions to produce homogeneous Ni_0.5_Mn_0.5−x_Sn_x_ alloys. When the annealing temperature was too cold or the annealing time was too short, ternary alloy film were not completely chemically homogenized. For example, both three-layer (*n* = 1) films annealed at 1073 K for 6 hours, and 1273 K for 1.5 hours illustrated: (1) a rugged sample surface, suggesting coexistence of multiple phases and a lack of equilibration (Fig. 5a), (2) heterogeneous cross-section images, illustrating that Mn, Sn, and Ni layers did not have sufficient time to completely interdiffuse (Fig. 6a), and (3) a lack of measurable calorimetric transformation signal in a homogeneous ternary alloy of that particular composition (Fig. 7). In contrast, if the annealing temperature was too high or the annealing time was too long (e.g., 6 hours at 1273 K), films lost significant overall mass, resulting in isolated grains, separated by large gaps (Fig. 5b). Increasing the annealing time at 1273 K from 1.5 to 6 hours resulted in an increase in mass loss during annealing from (4 to 65) wt%, which strongly impacted residual alloy composition, due to different equilibrium vapor pressures of different components.

To reduce the required annealing time, triple-layer (*n* = 1) films were replaced with multi-layer films (e.g., *n* = 3, 10, etc.), while maintaining a constant total thickness (Fig. 4c,d). This resulted in decreasing the characteristic timescale for diffusion for each layer. Annealing *n* = 3 films at 1273 K for 1.5 hours resulted in homogeneous surface topography, as well as cross-section images, suggesting complete inter-diffusion of each monatomic layer (Fig. 6b). This approach resulted in 4 wt% mass loss. Increasing the total number of layers (from *n* = 3 to *n* = 10) while maintaining a constant overall thickness, and annealing at 1273 K for 1 min (heated at 30 K/min from 25 to 1173 K to decrease time for oxygen exposure and at 10 K/min from 1173 to 1273 K) resulted in a homogeneous alloy with less than 0.25 wt% mass loss during annealing.

#### Compositional Control

**Figure 8:**
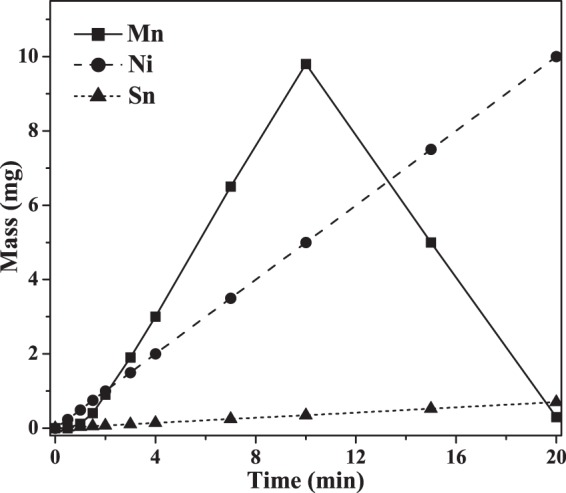
Deposition mass-time of Mn, Sn, and Ni at current densities of 80, 1, and 30 mA/cm^2^, respectively, for a working electrode area of 0.95 cm^2^.

Electrochemical deposition of thirty-layer films (*n* = 10) annealed at 1273 K for 1 min, resulted in compositionally homogeneous ternary alloys. Overall alloy compositions were controlled by adjusting the deposition time for each layer, normalized to retain constant overall film thickness. Required deposition times were calculated based on calibrated deposition rates of monatomic films, which were observed to behave fairly linearly for Sn and Ni over time and Mn after the second minute (Fig. 8). As previously mentioned, Mn deposition rate decreased after ten minutes due to manganese oxide coverage of the counter electrode.

We fabricated four 28.9 µm thick alloys with increasing Sn concentration, Ni_0.50_Mn_0.42_Sn_0.08_ (x = 0.08), Ni_0.50_Mn_0.10_Sn_0.10_ (x = 0.10), Ni_0.50_Mn_0.385_Sn_0.115_ (x = 0.115), and Ni_0.50_Mn_0.37_Sn_0.13_ (x = 0.13). EDS composition analyses were collected (Table 1), and were within 0.002 of the target compositions, illustrating effective control of overall composition by calibrated deposition time of individual elemental layers.

**Table 1:** Compositions of the target alloys (Ni_0.5_Mn_0.5−x_Sn_x_), determined by EDS analysis.

Target Sn	Ni	Mn	Sn
0.080	0.500	0.419	0.081
0.100	0.500	0.402	0.098
0.115	0.501	0.385	0.114
0.130	0.499	0.369	0.131

#### Phase purity

**Figure 9:**
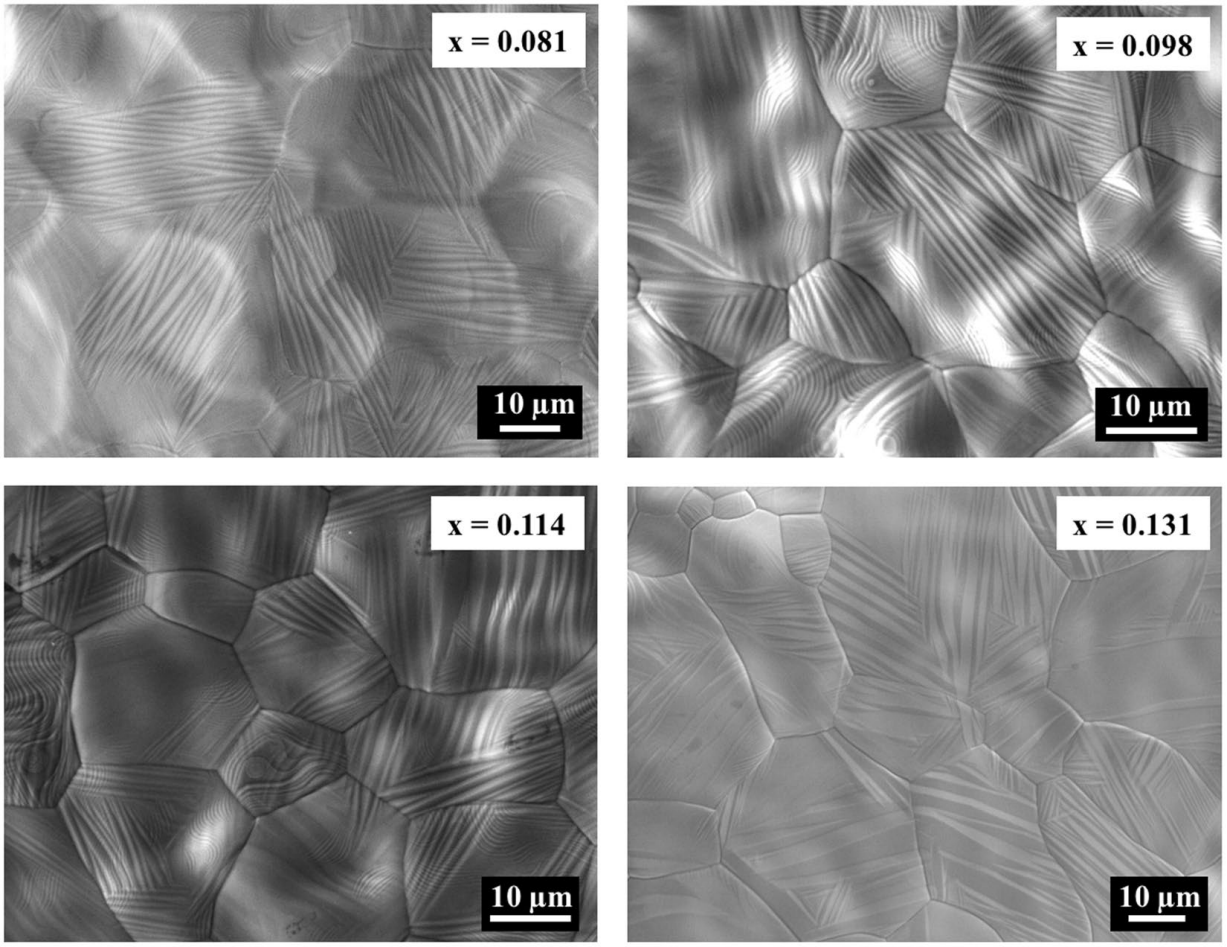
Microstructure of the alloys (Ni_0.5_Mn_0.5−x_Sn_x_, 0.081 ≤ x ≤ 0.131) under SEM.

Microstructures of annealed thick films were explored using SEM. All of the alloys illustrated twinning of martensitic variants expected for such alloys at room temperature (Fig. 9). The twins in the plates changed from a fine form to a broad form with the increase of Sn concentration, indicative of a transition from 14 *M* to 10 *M* or 4 *O* martensitic variants^46^. No phase separation was identified in the Sn concentration range 0.081 ≤ x ≤ 0.114. The alloy x = 0.131, exhibited coexistence of high temperature parent phase (*L*2_1_) and low temperature martensite phase due to the near-room temperature transformation in the alloy.

**Figure 10:**
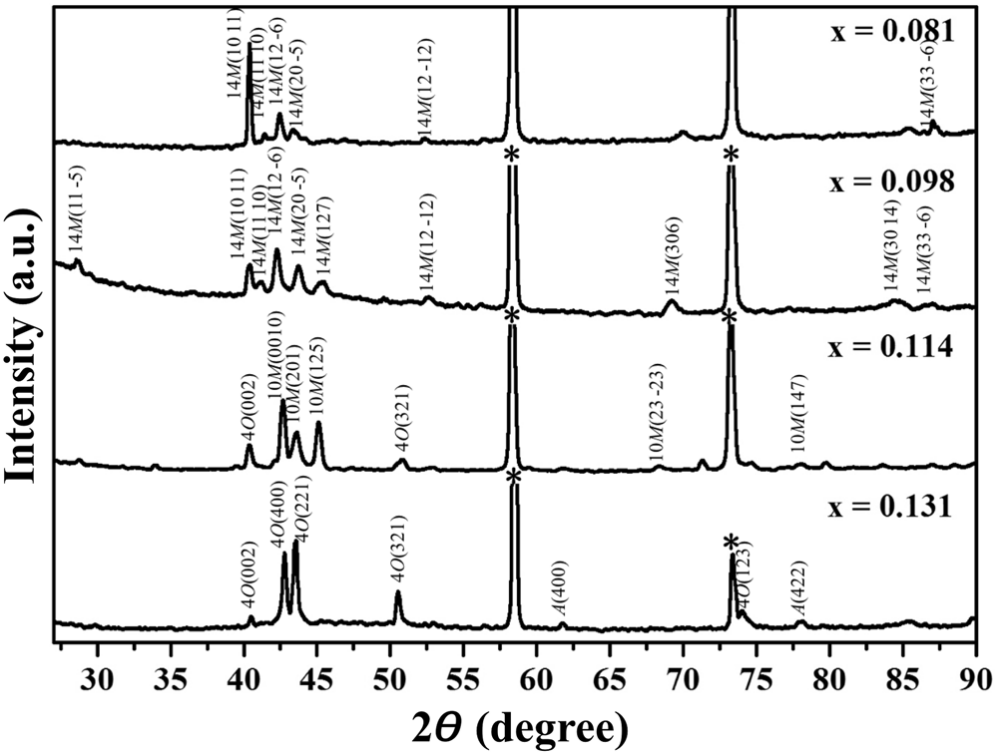
XRD patterns of the alloys (Ni_0.5_Mn_0.5−x_Sn_x_, 0.081 ≤ x ≤ 0.131) at room temperature (cubic W substrate reflections were marked with asterisks).

XRD analyses of annealed thick films on W substrate were performed to identify phases and crystal structures at room temperature. From top to bottom, the XRD patterns showed the alloys with x = 0.081, x = 0.098, x = 0.114, and x = 0.131, respectively, with indexed peaks corresponding to 14*M*, 10 *M*, 4*O*, and *L*2_1_ structures indicated by 14*M* (hkl), 10*M* (hkl), 4*O* (hkl), and A (hkl) (Fig. 10)^46,47^. At room temperature, the structure of alloy with x = 0.131 was identified as a mixture of four-layered orthorhombic (4*O*) structure (martensite) and *L*2_1_ cubic structure (austenite)^48^. The alloy with x = 0.081, x = 0.098, and x = 0.114 were martensitic. However, their crystal structures were different. The peaks of alloys with x = 0.081 and x = 0.098 corresponded with 14*M* monoclinic structure^46^. The alloy with x = 0.114 was identified as a mixture of 10*M* orthorhombic structure and 4*O* orthorhombic structure^47^. Thus, with Sn replacing Mn and the broad form twins replacing the fine form twins on the basis of Ni_0.5_Mn_0.5−x_Sn_x_, the crystal structures changed from 14*M* to 10*M*, and then to 4*O* and *L*2_1_.

## Discussion

### Phase Transformation Behavior

**Figure 11:**
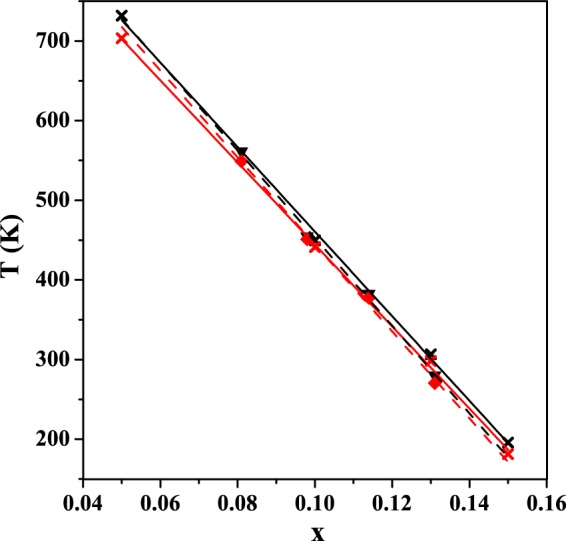
The comparison of exothermic (red dashed line with diamond) and endothermic (black dashed line with triangle) peak temperatures in annealed alloy films, against previously reported average martensitic (red solid line with ×), (*M*_s_ + *M*_f_)/2, and the reverse (black solid line with ×), (*A*_s_ + *A*_f_)/2, transformation temperatures for bulk alloys (Ni_0.5_Mn_0.5−x_Sn_x_) by linear regression^46,49,50^.

Exothermic and endothermic peak temperatures of annealed (*n* = 10) alloys identified from DSC curves were consistent with the martensitic and the reverse transformations previously reported in bulk alloys (Fig. 11)^46,49,50^. Here, we compare the exothermic and endothermic peak temperatures of the alloy films against previously reported average temperatures of the transformation on cooling, *T*_M_ = (*M*_s_ + *M*_f_)/2, and on heating, *T*_A_ = (*A*_s_ + *A*_f_)/2, where *M*_s_ and *M*_f_ refer to martensite start temperature and martensite finish temperature, and *A*_s_ and *A*_f_ refer to austenite start temperature and austenite finish temperature. The previous relationships between average transformation temperatures and compositions were *T*_M_ = 959.6–5154.2*X* for cooling and *T*_A_ = 991.5–5311.9*X* for heating, where x represented stoichiometric ratio in Ni_0.5_Mn_0.5−x_Sn_x_. Our relationships between alloy film transformation peak temperatures and compositions were *T*_M_ = 990.4–5462.1*X* for cooling and *T*_A_ = 1002.9–5506.9*X* for heating. The difference between parameters in previous relationships and our relationships was less than 6%. The deviations of exothermic and endothermic peak temperatures were within 16 K of previously reported average temperatures. The near-room temperature martensitic phase transformation of alloy with x = 0.131 explained the phase coexistence of 4*O* and *L*2_1_ structures during XRD measurement. Smaller atomic layers (*n* = 10 vs. *n* = 3) resulted in sharper transformation widths (10 to 15 K vs. >50 K), indicative of less compositional heterogeneity within the film (Fig. 7).

Due to the sensitivity of transformation temperatures with composition, repeatability of film compositions was assessed by examining the transformation temperature of 4 identical prepared alloy films (Fig. S5). Four *n* = 10 alloys (Ni_0.500_Mn_0.419_Sn_0.081_) were deposited and annealed under identical conditions, and measured by DSC. The expected bulk exothermic (544.5 K) and endothermic (562.5 K) peak temperatures were obtained from linear fit to previously reported bulk alloy data (Fig. 11). The observed average peak temperatures for the repeated films were 547.9 K on cooling and 560.1 K on heating. The standard deviations of these 4 identical alloy films were σ = 3.4 K on cooling and σ = 2.4 K on heating, confirming good repeatability of film compositions and film transformation behavior.

### Alloy Film Microstructure

**Figure 12:**
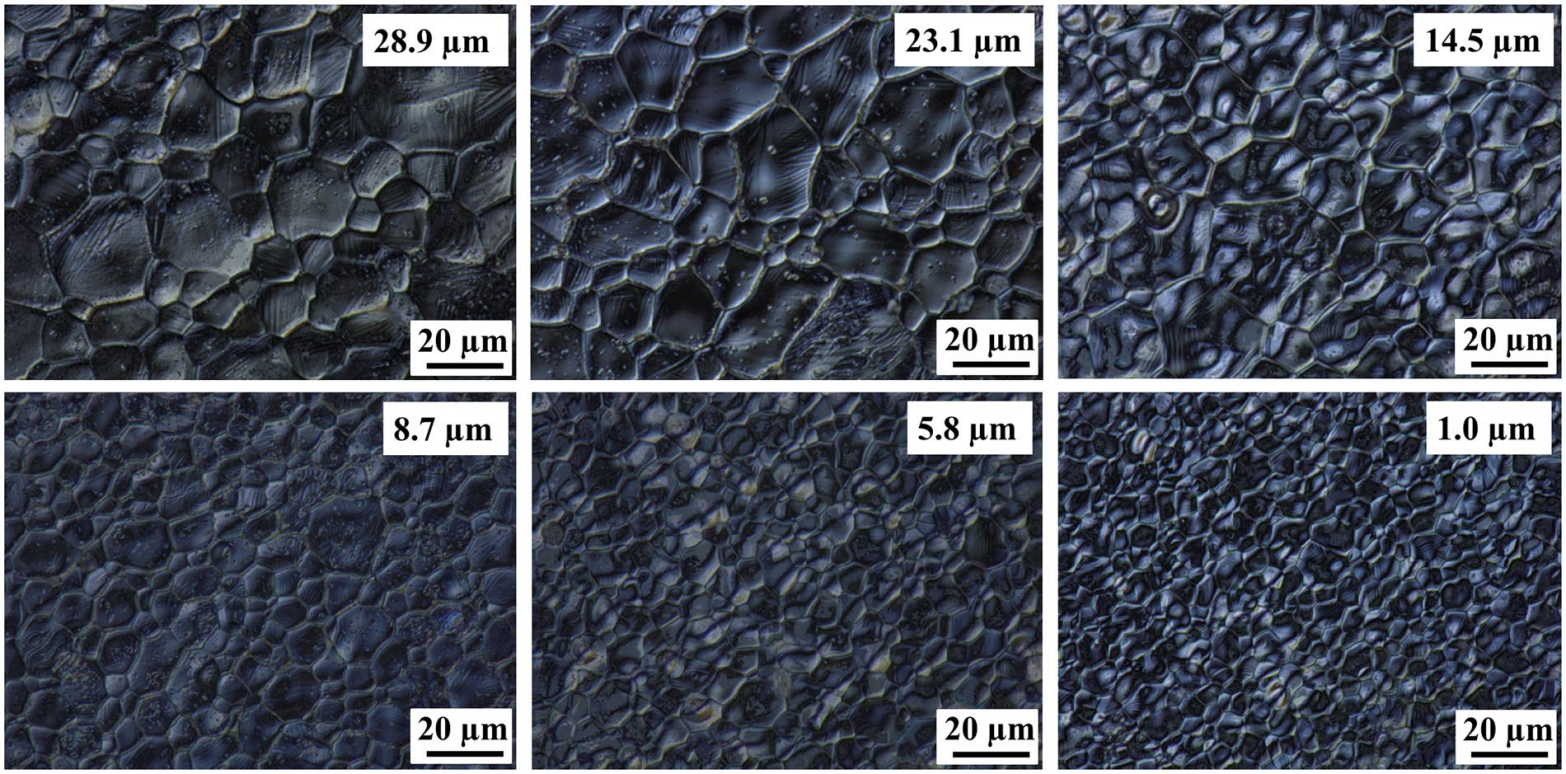
Surface micrographs of the alloys Ni_0.500_Mn_0.419_Sn_0.081_, with decreasing thicknesses under optical microscope.

Alloy films of different thicknesses (1.0 to 28.9 µm) but with constant elemental layer thicknesses were deposited in order to explore the relation between film thickness and the grain size. These included the 28.9 µm W//(Mn/Sn/Ni)_10_, the 23.1 µm W//(Mn/Sn/Ni)_8_, the 14.5 µm W//(Mn/Sn/Ni)_5_, the 8.7 µm W//(Mn/Sn/Ni)_3_, the 5.8 µm W//(Mn/Sn/Ni)_2_, and the 1.0 µm W//(Mn/Sn/Ni) with the composition x = 0.081 (Fig. 12). The alloys showed surface deformation consistent with martensite twining in thick films (28.9 to 14.5 µm). It was difficult to discern the martensitic structure when the thicknesses were thinner than 8.7 µm.

**Figure 13:**
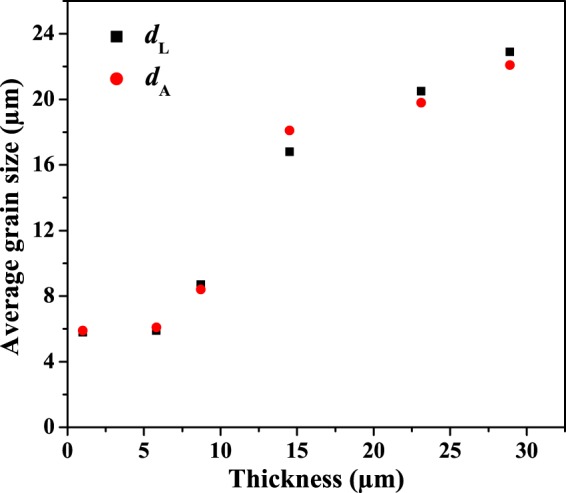
Grain sizes of different thickness alloys Ni_0.500_Mn_0.419_Sn_0.081_, based on intercept or areal, counting methods.

Alloy grain sizes were analyzed by linear and areal techniques using the following equations, where *d*_L_ was the average grain size based on intercepts and *d*_A_ was the average grain size based on areas (Fig. 13)^51^.

2 $${d}_{{\rm{L}}}=\frac{2\times 3L}{4{N}_{{\rm{L}}}}$$

3 $${d}_{{\rm{A}}}=2\sqrt{\frac{3A}{2\pi {N}_{{\rm{A}}}}}$$

4 $${N}_{{\rm{A}}}={N}_{{\rm{whole}}}+\,\frac{{N}_{{\rm{edge}}}}{2}$$

*L* was the length of line, and *A* was the inspected area. *N*_*L*_ was the number of intercepts. *N*_whole_ and *N*_edge_ were the numbers of the whole grains and edge grains in the area. *N*_A_ was the effective number of grains. The differences between *d*_L_ and *d*_A_ were less than 8% (Table S1), which verified that both *d*_L_ and *d*_A_ decreased with the reducing of the alloy film thicknesses. Average grain size was approximately proportional to the film thickness (Fig. 13). Grains in a poly-crystalline film span the thickness of the film and their surface traces can be approximated by circular arcs. When grain size becomes large enough and the magnitude of the surface angle is less than a critical value, grain growth must stop^52^. This growth mechanism tends to result in the observed proportionality between film thickness and grain size.

### Optical transformation

**Figure 14:**
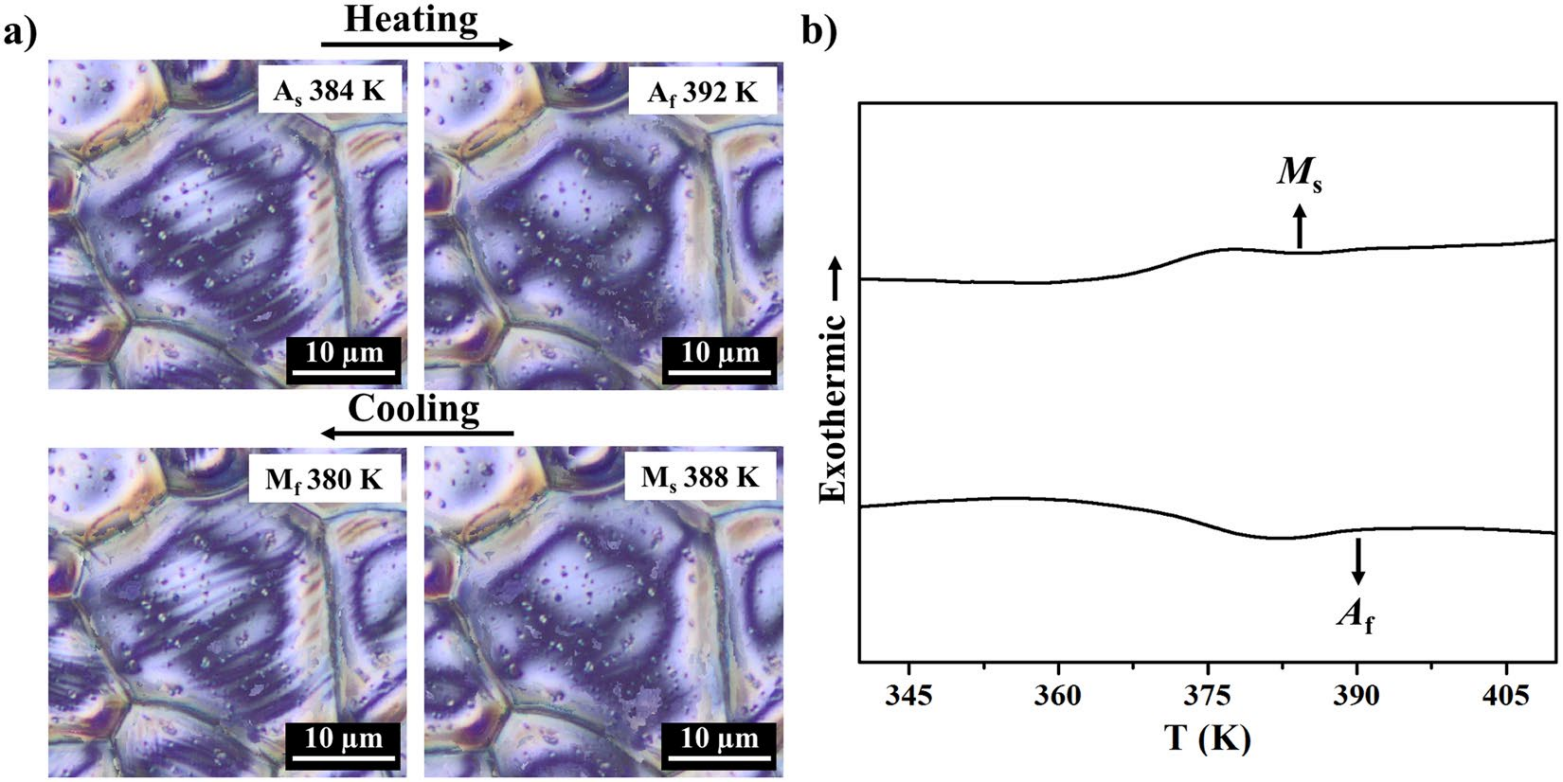
(**a**) Surface micrographs and (**b**) DSC measurement of the alloys Ni_0.500_Mn_0.385_Sn_0.115_ during heating and cooling.

Structural phase transitions of individual grains on the film Ni_0.500_Mn_0.385_Sn_0.115_ are investigated optically while the sample is heated or cooled through the phase transition. A DIC prism was used to introduce contrast due to small difference in optical path length, allowing for observation of surface deformation associated with the martensitic transformation. We observed that the transformation behavior of each grain was independent from neighboring grains. The austenite finish temperature (*A*_f_, 392 K) and martensite start temperature (*M*_s_, 387 K) for a particular grain were recognized when the last twin disappeared completely and the first twin formed abruptly (Fig. 14a). The film *M*_s_ (390 K) and *A*_f_ (384 K) were measured by DSC (Fig. 14b). After comparing the micro-scale observation for one particular domain (Fig. 14a) and the macroscopic aggregate transformation (Fig. 14b), we found that the widths of transformation peaks in each grain, ((*M*_s_ − *M*_f_) or (*A*_s_ − *A*_f_), 8 K), were smaller than widths in macroscopic transformation peaks (>15 K), but the hysteresis, (*A*_f_ − *M*_s_) was similar. Thus, deposition of alloy films as described in this work is an effective way to analyze the size and thickness dependent hysteresis by a statistical analysis of populations of grains in the film. We will report the related research in the future.

## Conclusions

A fabrication approach for Ni_0.5_Mn_0.5-x_Sn_x_ Heusler alloys by multi-layer electrochemical deposition on smooth W substrates was demonstrated. Mn, Ni, and Sn monatomic films with different thicknesses were deposited under different current densities, resulting in dense, smooth films. Ni_0.5_Mn_0.5−x_Sn_x_ alloys were deposited by repeated stacking of 3n-layer monatomic films, in which individual layer thickness was adjusted based on deposition time. By increasing the total number of layers to n = 10, thereby decreasing the thickness of each individual layer, the necessary annealing temperatures and times were reduced, allowing for chemical homogeneity, while minimizing mass loss and oxidation during annealing. We demonstrate this technique on four alloy compositions, followed by post-deposition annealing at 1273 K. These films proved to be compositionally homogeneous, as determined by the repeatability of transformation peak temperatures, which also closely match bulk transformation temperatures. A positive correlation between alloy film thicknesses and grain sizes was observed. Correspondence between overall film transformation temperatures and local transformation temperatures of individual grains is demonstrated by optical transformation. This deposition approach combined with microscopic observations of optical transformations allows for a platform for investigation of the role of dimensionality in reversible martensitic phase transformation in multifunctional alloy films.

## Methods

Electrochemical deposition was performed with a Biologic SP-150 potentiostat using a standard three-electrode configuration. The counter electrode for Sn and Mn deposition was platinum-coated titanium mesh with a surface area of 10 cm^2^ (American Elements). The counter electrode for Ni deposition was nickel wire with a surface area of 10 cm^2^ (Alfa Aesar). The reference electrode for Mn and Sn deposition was a standard Ag/Ag_2_SO_4_ electrode from Koslow Scientific Company. The reference electrode for Ni deposition was a Ag/AgCl electrode. Circular Ni, Sn, and Mn films (0.95 cm^2^) were deposited on 0.05 mm thick, high purity tungsten substrates (Alfa Aesar, 99.95% pure) after mechanically polished down to 1 µm grit size. The composition of the Mn deposition solution was 0.59 M MnSO_4_·H_2_O and 1 M (NH_4_)_2_SO_4_^40^. The composition of the Ni deposition solution was 0.2 M NiSO_4_·6H_2_O_,_ 0.3 M NiCl_2_·6H_2_O_,_ and 0.5 M H_3_BO_3_^53^. The composition of the Sn deposition solution was 0.1 M SnSO_4_ and 0.2 M C_6_H_5_Na_3_O_7_^54^. Primarily deposition from sulfate baths was chosen to minimize anion cross-contamination during the multi-pot electrochemical deposition process. Furthermore, because of the multi-pot technique employed in this study, we adjusted the solution pH in the range of 3 to 4 by the addition of sulfuric acid and sodium hydroxide in order to maintain similar pH in different baths. All of the solutions used deionized water at 298 K.

Mn cyclic voltammetry was performed from (2 to −2) V at a scan rate of 50 mV/s. Ni cyclic voltammetry was performed from (0.5 to −1.1) V at a scan rate of 50 mV/s. Sn cyclic voltammetry was performed from (−0.45 to −1.6) V at a scan rate of 10 mV/s. Mn, Ni, and Sn films were deposited under constant current densities (chronopotentiometry). Deposition rate was calculated directly from measured mass changes, normalized by bulk metal densities and deposition areas. A two-step sequence was applied in Mn deposition for improving film smoothness and coverage area, which consisted of a pre-conditioning step and a deposition step^39^. Manganese was deposited for 1 min, after which the power supply was turned off for 10 seconds, resulting in dissolution of this initial film. Subsequently, a second layer of Mn was deposited to the desired thickness. To protect the samples from oxidation, films were immersed into acetone immediately after depositing.

Multi-layer films were annealed in a high temperature tube furnace (OTF-1200X-S; MTI Corporation) with argon (purity 99.999%) −5% hydrogen forming gas at 101 kPa. An in-line dessicant and oxygen getter (Restek) was used to trap oxygen and water to purify forming gas. During annealing, samples were placed in an alumina boat wrapped in a Ti envelope, and several porous titanium sponges (Alfa Aesar) were placed immediately upstream of the sample to absorb the residual oxygen.

Scanning Electron Microscopy (SEM) was performed on a Tescan Vega 3 equipped with an Oxford energy dispersive spectrometer (EDS) X-act at an accelerating voltage of 20 keV, in secondary electron imaging mode. Differential Scanning Calorimetry (DSC) was performed on a TA Instrument Q2000 DSC in Al pans under a nitrogen atmosphere using heating and cooling rates of 10 K/min. X-ray diffraction (XRD) measurements were performed with a Bruker D8 Advance diffractometer with Cu Kα radiation and a parallel incident beam. Topographic characterizations of as-received and mechanically polished W were carried out in a Bruker Dimension Icon AFM. Reported surface roughnesses represent areal root mean squared roughness values over a 1 μm^2^ area. Optical observations of phase transformation were carried out on a BX53M Olympus upright microscope equipped with a differential interference contrast (DIC) prism and a Linkam LTS 120 temperature controlled microscope stage-control ±0.1 K, calibrated with alloys with known melting points.

### Data availability

The datasets generated and analyzed during the current study are available from the corresponding author on reasonable request. All data generated or analyzed during this study are included in this published article and its Supplementary Information file.

## Electronic supplementary material

Supplementary Information
