# Supplementary material for: Thick Film Ni0.5Mn0.5−xSnx Heusler Alloys by Multi-layer Electrochemical Deposition
Source: Sci Rep. 2018 Aug 9;8:11931. doi: 10.1038/s41598-018-29628-8 (PMC6085326; doi:10.1038/s41598-018-29628-8)
Supplement: Supplementary file 1 — Supplementary Information [file 41598_2018_29628_MOESM1_ESM.docx]

# Thick Film Ni_0.5_Mn_0.5-x_Sn_x_ Heusler Alloys by Multi-layer Electrochemical Deposition

Yijia Zhang^1^

Patrick J. Shamberger^1^

[1] Department of Materials Science and Engineering

Texas A&M University

College Station, TX, 77843, USA

Phone: 979-458-1086

Fax: 979-862-6835

E-mail: patrick.shamberger@tamu.edu


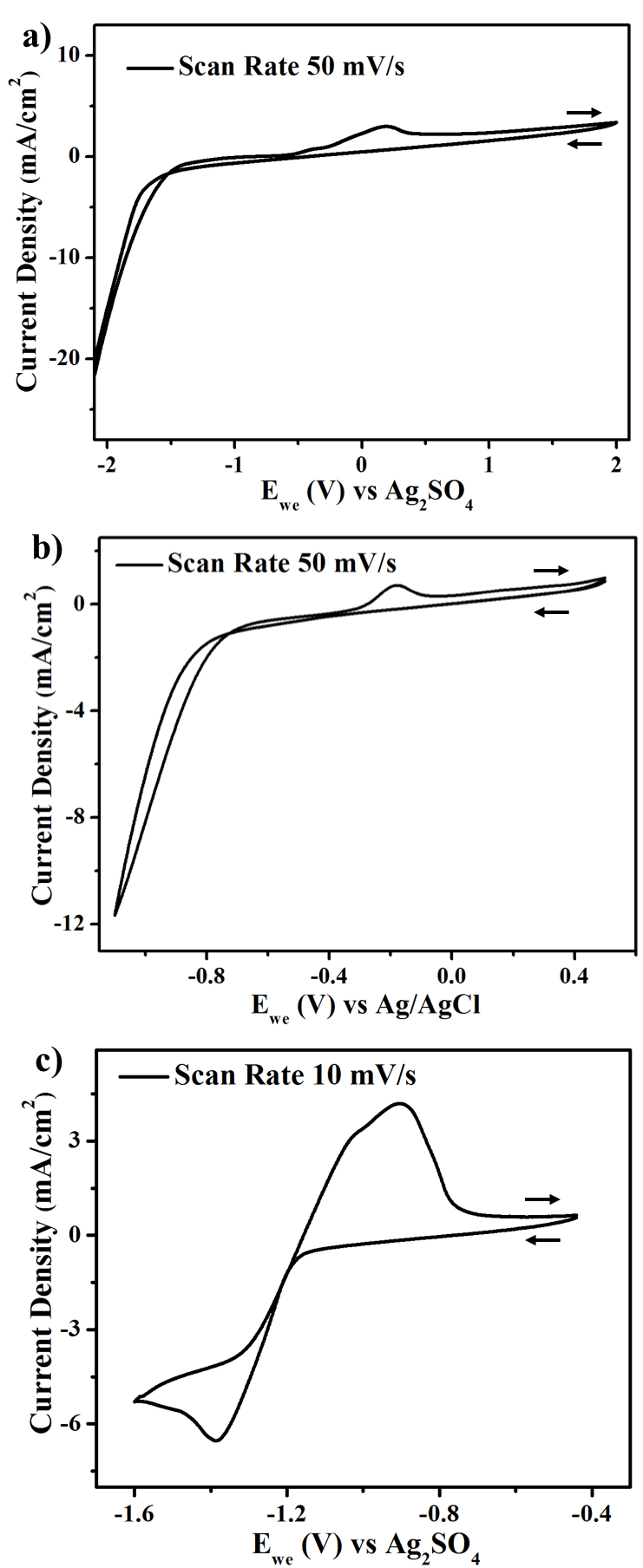


Fig. S1. Cyclic voltammetry of a) Mn in 0.6 M MnSO_4_·H_2_O and 1 M (NH_4_)_2_SO_4_ at scan rate of 50 mV/s, b) Ni in 0.2 M NiSO_4_·6H_2_O, 0.3 M NiCl_2_·6H_2_O, and 0.5 M H_3_BO_3_ (so-called Watts bath) at scan rate of 50 mV/s, and c) Sn in 0.1 M SnSO_4_ and 0.3 M C_6_H_5_Na_3_O_7_ aqueous solution at scan rate of 10 mV/s.


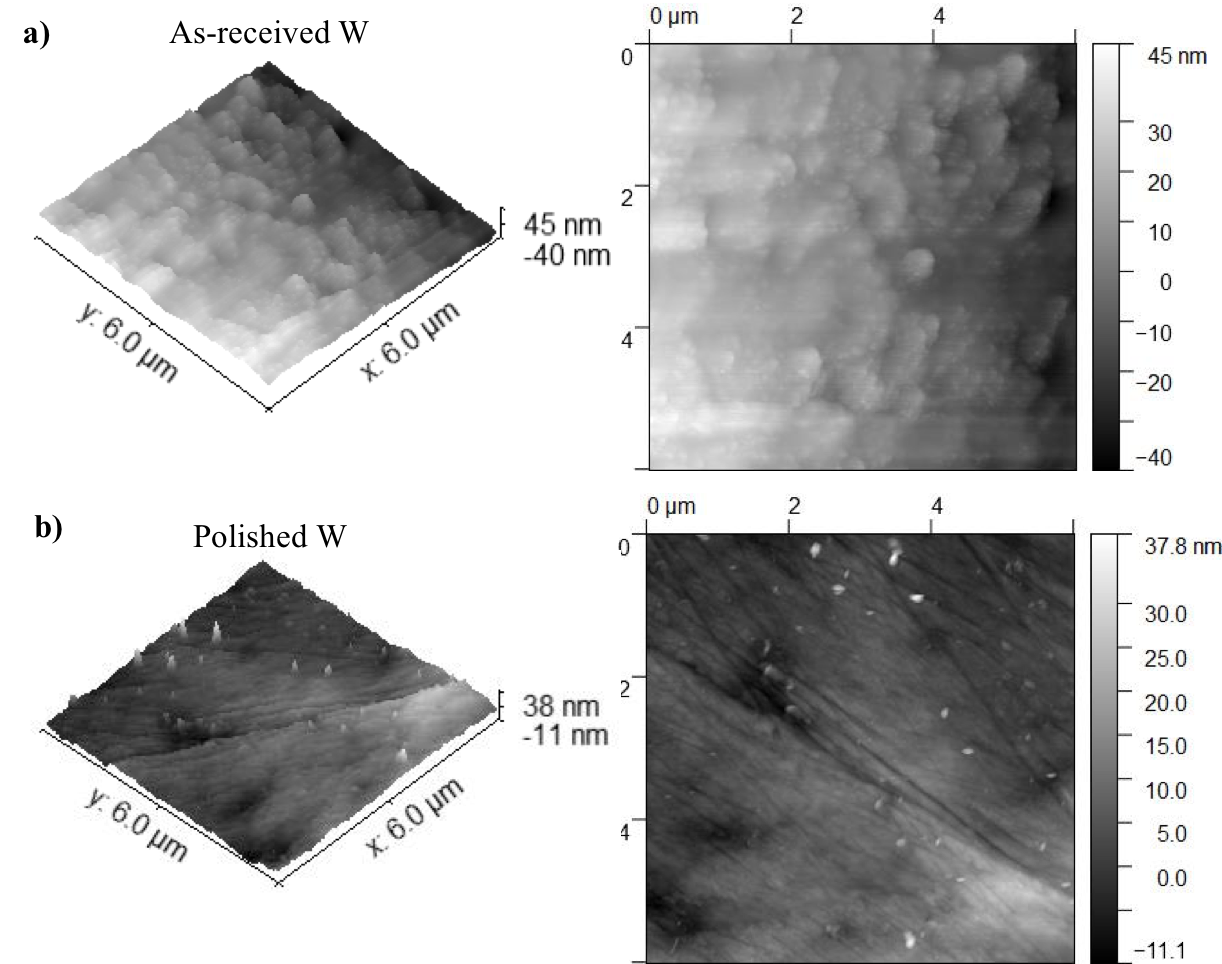


Fig. S2. Surface topography of a) as-received W (S_q_ = 15.9 ± 2.0 nm) and b) mechanically polished W (S_q_ = 5.3 ± 0.9 nm), as observed using AFM.


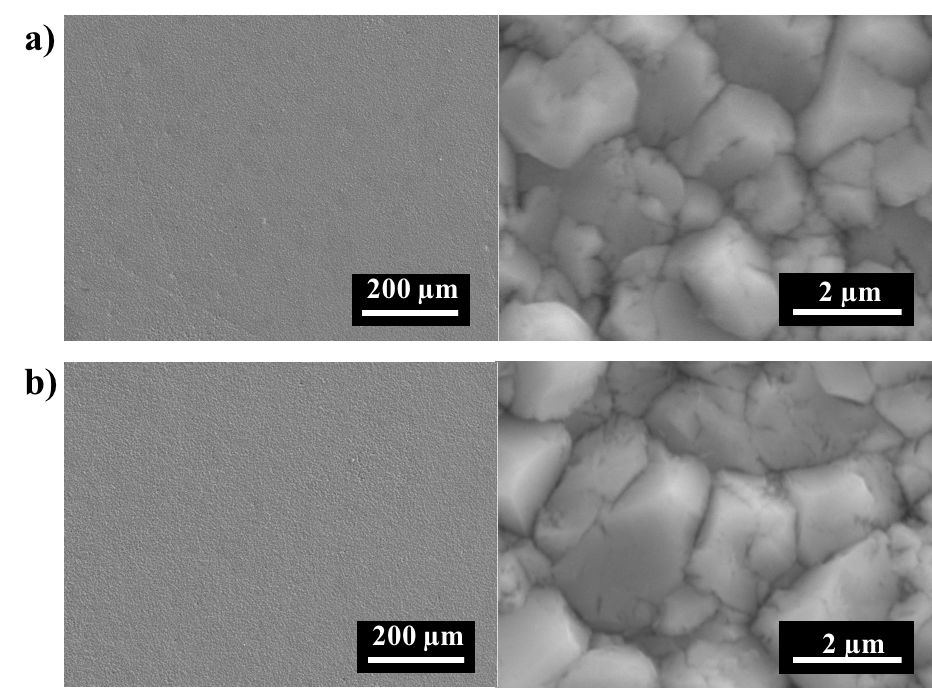


Fig. S3. 2.5 μm thick Mn films deposited from 0.6 M MnSO_4_·H_2_O and 1 M (NH_4_)_2_SO_4_ aqueous solutions a) without NH_4_OH, and b) with 1M NH_4_OH.


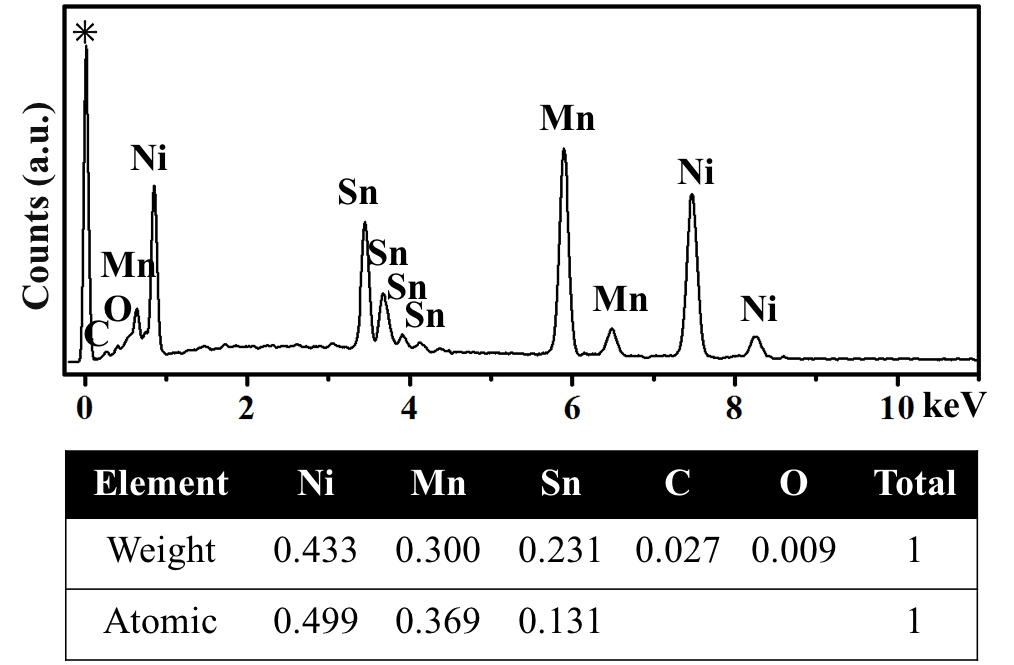


Fig. S4. Ni_0.50_Mn_0.37_Sn_0.13_ cross-section composition analysis of nine-layer films by EDS.


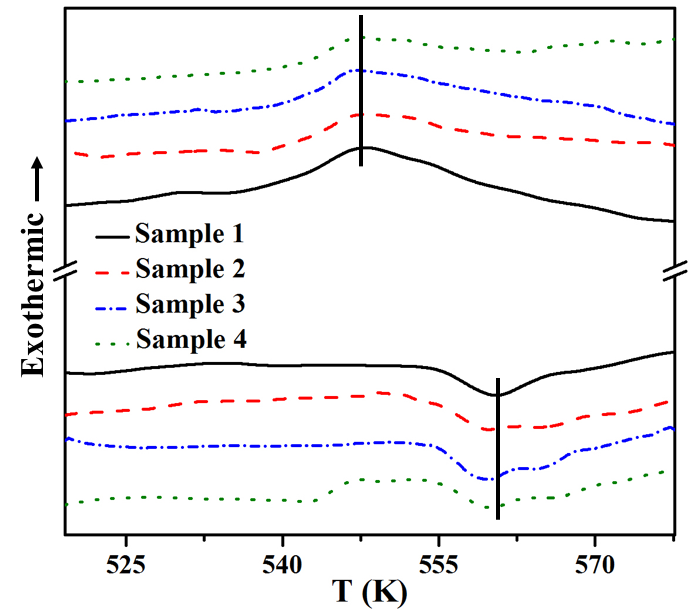


Fig. S5. The repeatability test of thirty-layer film annealed alloys (Ni_0.50_Mn_0.419_Sn_0.081_) by DSC, for four independently deposited and annealed alloys. Due to small sample mass, DSC signals are fairly weak.

Table S1. The grain sizes analyses of different thickness alloys Ni_0.50_Mn_0.419_Sn_0.081_ (x = 0.081) based on line intercept method, *d_L_*, or counting statistics within a defined circular area, *d_A_*.

| **Thickness (µm)** | **28.9** | **23.1** | **14.5** | **8.7** | **5.8** | **1.0** |
| --- | --- | --- | --- | --- | --- | --- |
| *d_L_* (µm) | 22.9 | 20.5 | 16.8 | 8.7 | 5.9 | 5.8 |
| *d_A_* (µm) | 22.1 | 19.8 | 18.1 | 8.4 | 6.1 | 5.9 |
